# Predictive value of ventriculo-arterial coupling for hypotension after induction of anaesthesia: a prospective observational cohort study

**DOI:** 10.1186/s40635-026-00899-0

**Published:** 2026-04-21

**Authors:** Hong Chinh Le, Chau Bich Ha Tran, Thi Thu Trang Nguyen, Ton Ngoc Vu Phan, Duc Nam Nguyen, Trung Hieu Duong, Tran Huy Nhat Phung, Huu Khanh Trinh Dong, Yvon L. J. M. Deryck, Minh Khoi Le

**Affiliations:** 1 Department of Critical Care Medicine, Emergency and Clinical Toxicology, School of Medicine, University of Medicine and Pharmacy at Ho Chi Minh City, 217 Hong Bang street, Cho Lon Ward, Ho Chi Minh City, 72714 Vietnam; 2 Department of Anaesthesia, University Medical Center Ho Chi Minh City, Ho Chi Minh City, Vietnam; 3 Echocardiography Laboratory, University Medical Center Ho Chi Minh City, Ho Chi Minh City, Vietnam; 4 Bordeaux Population Heath, Institut National de la Santé et de la Researche Médicale (INSERM) U1219, Université de Bordeaux, 146 Rue Leo Saignat, 33000 Bordeaux, France; 5 Department of Anaesthesia, Ghent University Hospital, Ghent, Belgium

**Keywords:** Post-induction hypotension, Ventriculo-arterial coupling, Effective arterial elastance, Left ventricular end-systolic elastance, Echocardiography

## Abstract

**Background:**

Post-induction hypotension (PIH) is associated with acute perioperative organ injury. We quantified the added value of echocardiographic parameters and ventriculo-arterial coupling (VAC)-related variables for the prediction of PIH.

**Methods:**

A prospective observational cohort study conducted between July 2023 and November 2024 enrolled adults undergoing elective non-cardiac surgery. The ventriculo-arterial coupling index (*E*_a_/*E*_es_) was derived pre-operatively using transthoracic echocardiography combined with non-invasive blood pressure measurements. PIH was defined as the first occurrence of mean arterial pressure < 65 mmHg between anaesthesia induction and surgical incision. Predictors were analysed using a generalised additive model to account for potential non-linear associations. Incremental prognostic value was assessed using the fraction of new information (FNI), a reclassification-based metric, and changes in the area under the receiver operating characteristic curve (AUC).

**Results:**

PIH occurred in 161/405 patients (39.8%). An *E*_a_/*E*_es_ ratio > 1 was associated with PIH (OR 2.95; 95% CI 1.08–8.03; *p* = 0.034). The clinical model showed an AUC of 0.720 (95% CI 0.669–0.771). The addition of echocardiographic parameters increased the AUC to 0.768 (95% CI 0.720–0.816; Holm-adjusted* p* = 0.018) and provided 37% FNI (Holm-adjusted* p* = 0.199). The incorporation of VAC-related variables further increased the AUC to 0.785 (95% CI 0.739–0.831; Holm-adjusted* p* = 0.002) and yielded 46% FNI (Holm-adjusted* p* = 0.030). Compared with the clinical–echocardiographic model, incorporation of VAC-related variables provided an additional 14% FNI (Holm-adjusted* p* = 0.018) without a statistically significant AUC increase (0.017; 95% CI −0.004–0.039; Holm-adjusted* p* = 0.199).

**Conclusion:**

PIH was common and was independently associated with impaired VAC, as reflected by an *E*_a_/*E*_es_ ratio > 1. Incorporation of echocardiographic parameters improved the discriminatory performance of a clinical prediction model, and the further addition of VAC-related variables provided meaningful improvement in risk reclassification, despite only modest gains in overall discrimination.

**Supplementary Information:**

The online version contains supplementary material available at 10.1186/s40635-026-00899-0.

## Introduction

Hypotension during anaesthesia is a common haemodynamic disturbance and has been linked to serious complications such as myocardial injury, acute kidney injury, myocardial infarction, and even death [1, 2]. Post-induction hypotension (PIH) occurs between anaesthesia induction and skin incision [3, 4] and represents a vulnerable period for haemodynamic compromise. The incidence of PIH varies according to the mean arterial pressure (MAP) threshold used to define hypotension, occurring in 53% of patients at a threshold of 65 mmHg [5], and in 34% at a threshold of 55 mmHg [4]. Despite its brief duration, PIH is associated with an increased incidence of acute kidney injury [5] and stroke [6].

Several factors have been identified as risk factors for PIH, including advanced age, comorbidities, emergency surgery, hypovolemia, and the use of agents such as propofol, high-dose opioids, or renin–angiotensin–aldosterone system inhibitors [7]. Echocardiographic findings, such as regional wall motion abnormalities and indices of diastolic dysfunction, have also been linked to an increased risk for PIH [8].

When hypotension is caused by vasodilation, the left ventricle must generate sufficient flow to maintain arterial pressure. Cardiac mechanical efficiency, defined as the ratio of stroke work to myocardial oxygen consumption [9], may therefore represent a valuable physiological marker for predicting PIH. However, cardiac mechanical efficiency is difficult to measure accurately in both experimental and clinical settings.

The pressure–volume plane provides a framework for characterising the left ventricle by end-systolic elastance (*E*_es_) and the arterial system by effective arterial elastance (*E*_a_) [10, 11]. This approach conceptualises the anatomical connection between the left ventricle and arterial tree as a functional interaction between two elastic chambers, referred to as ventriculo-arterial coupling (VAC). VAC is quantified by the *E*_a_/*E*_es_ ratio [12]. Burkhoff and Sagawa demonstrated that cardiac mechanical efficiency depends on the *E*_a_/*E*_es_ ratio, with a value approximating 1 representing optimal VAC in terms of stroke work [9]. The *E*_a_/*E*_es_ ratio can be determined at the bedside using transthoracic echocardiography (TTE) and non-invasive blood pressure (NIBP) measurements [13], enabling integration of VAC assessment into routine clinical practice [12].

The VAC framework has proven valuable for predicting fluid responsiveness and the haemodynamic effects of norepinephrine in critically ill patients [14, 15] and has contributed to the understanding of the cardiovascular effects of anaesthetic agents [16, 17]. Yildirim et al*.* demonstrated that *E*_a_ is a reliable predictor of hypotension following induction of general anaesthesia [18]. In this context, the VAC framework may offer a novel approach for predicting PIH and enhancing pre-operative risk stratification. We therefore hypothesised that VAC determinants, particularly an *E*_a_/*E*_es_ ratio > 1, would improve prediction of PIH beyond that achieved with clinical and conventional echocardiographic measurements.

## Methods

### Study participants

This study was approved by the Institutional Review Board of the University Medical Center Ho Chi Minh City, Vietnam (No. 32/GCN-HDDD, 18 May 2023) and registered at ClinicalTrials.gov (NCT05969886). Written informed consent was obtained from all participants.

Inclusion criteria comprised haemodynamically stable adults (≥ 18 years) scheduled for elective non-cardiac, non-obstetric and non-gynaecologic surgery under general anaesthesia with endotracheal intubation. Eligible patients were classified as American Society of Anesthesiologists (ASA) physical status I–IV and had undergone TTE within 48 h before induction.

Exclusion criteria included uncorrected congenital heart disease, moderate-to-severe valvular disease or pulmonary hypertension, arrhythmias, an anticipated difficult airway, and known allergies to anaesthetic agents.

### Study design

This prospective observational cohort study was conducted at the University Medical Center Ho Chi Minh City between July 2023 and November 2024.

### Sample size estimation

The sample size was calculated to detect PIH risk with an expected area under the receiver operating characteristic curve (AUC) of 0.70, assuming improved model performance after the addition of VAC-related variables (continuous *E*_a_ and *E*_es_, and an *E*_a_/*E*_es_ ratio > 1) to the base model. With an *α* of 0.05, a power of 0.90, and a 2:1 ratio of non-PIH to PIH patients, a total of 93 participants (62 non-PIH, 31 PIH) were required. For estimation of PIH incidence [5] (*p* = 0.53, *d* = 0.05), the minimum required sample size was 383. Allowing for a 5% attrition rate, the final target enrolment was at least 403 patients.

### Data collection before anaesthesia

Clinical and laboratory data, including comorbidities and pre-operative medications, were extracted from pre-operative assessments and electronic medical records in accordance with institutional protocol. Pre-operative TTE was performed by credentialed cardiologists with ≥ 5 years of experience using a Philips EPIQ 7C system (Philips Healthcare, Andover, MA, USA) with X5-1 and eL18-4 probes for cardiac and vascular imaging, respectively. Measurements followed American Society of Echocardiography guidelines [19, 20] and included left ventricular ejection fraction (EF) assessed using the Simpson biplane method, stroke volume (SV), velocity–time integral (VTI), and left ventricular outflow tract diameter (LVOT). Additional measurements included ascending aortic diameter, pre-ejection period (PEP), total systolic period (TSP), and estimates of aortic mechanical properties. Systolic (SAP) and diastolic (DAP) blood pressures were recorded non-invasively at the brachial artery during the scan.

*E*_a_ was calculated as *E*_a_ = 0.9×(SAP/SV), representing end-systolic pressure divided by the left ventricular stroke volume. *E*_es_ was calculated as the change in left ventricular pressure over the change in volume at end-systole according to the formula:

$$E_{es} { = }\,\left[ {{\mathrm{DAP}}{ - }\left( {{\mathrm{End}}_{{\mathrm{(est)}}} \times {\mathrm{SAP}}\, \times \,{0}{\mathrm{.9}}} \right)} \right]{/}\left( {{\mathrm{End}}_{{\mathrm{(est)}}} \times {\text{ SV}}} \right),$$

where End_(est)_ is the estimated normalised ventricular elastance at the onset of ejection [13]. *E*_a_, *E*_es_, and the *E*_a_/*E*_es_ ratio were derived using iElastance^®^ software, with SAP, DAP, EF, SV, PEP, and TSP entered as input variables.

### Anaesthesia induction and measurements

Anaesthesia was administered in accordance with the Department of Anaesthesia’s standard protocol. Peripheral intravenous access was established, and standard monitoring included electrocardiography, NIBP (measured every 2 min), arterial oxygen saturation, end-tidal CO_2_, and bispectral index (BIS).

Induction of general anaesthesia was initiated with fentanyl (1–3 µg/kg), followed by propofol titrated in 40 mg increments every 10–20 s to achieve a BIS value of 50. Rocuronium (0.6 mg/kg) was administered to facilitate tracheal intubation. Patients were mechanically ventilated with an air–oxygen mixture (40% O_2_) to maintain normocapnia. An additional 20 mg bolus of propofol was provided if BIS exceeded either 50 before intubation or 60 during intubation [21, 22]. Anaesthesia was maintained with sevoflurane, targeting a BIS of 40–60.

Blood pressure was also recorded immediately before and after intubation. PIH was defined as the first recorded MAP below 65 mmHg between induction and skin incision. Management of hypotension was left to the discretion of the attending anaesthetist.

### Statistical analysis

Statistical analyses were conducted using R (version 4.4.2). Data normality was assessed using the Shapiro–Wilk test. Continuous variables with normal distribution were compared using Welch’s *t*-test (mean ± SD), whereas non-normally distributed variables were compared using the Wilcoxon rank-sum test (median [IQR]). Categorical variables were compared using the *χ*^2^ test or Fisher’s exact test when expected cell counts were < 5. Binary variables with fewer than five observations per group were excluded from regression analyses and missing global longitudinal strain (GLS) values (< 5%) were imputed using the median.

Associations between candidate risk factors and PIH were evaluated using a generalised additive model (GAM) [23] with categorical predictors expressed as odds ratios and continuous predictors modelled non-linearly.

Predictive performance was assessed for three models: (1) M_0_, comprising clinical variables selected via least absolute shrinkage and selection operator (LASSO) regression [24]; (2) M_echo_, which added echocardiographic measurements; and (3) M_full_, which additionally included VAC-related variables. Added value was assessed by the fraction of new information (FNI), a reclassification-based metric, based on likelihood-ratio tests (see Supplementary Method S1 for details of the calculation) and by change in AUC (ΔAUC), evaluated using the DeLong test. All reported *p*-values were adjusted for multiple testing using Holm’s method to control the family-wise error type I rate at 0.05 [25]. Variable contributions in M_full_ were expressed as the percentage of relative explained variance in PIH risk, with 95% confidence intervals estimated from 2000 bootstrap samples.

## Results

### Patient characteristics, clinical and echocardiographic measurements

**Fig. 1:**
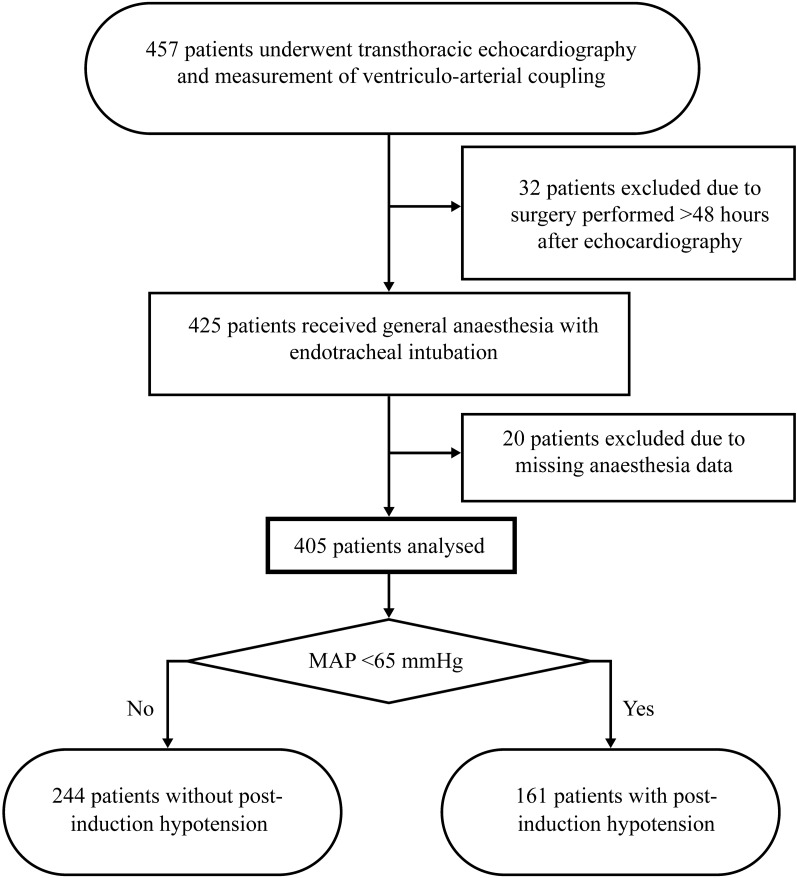
Flowchart of patient selection for post-induction hypotension analysis. *MAP* mean arterial pressure

A total of 405 patients who met the inclusion and exclusion criteria were included in the analysis (Fig. 1). The incidence of PIH, defined as the first recorded MAP below 65 mmHg, was 39.8%. The full multivariable model (M_full_) combined six clinical predictors selected by LASSO regression with 19 echocardiographic measurements and three VAC-related variables.

Compared with patients without PIH, those with PIH were older (median, 59 vs. 55 years; *p* = 0.003) and had a higher prevalence of ASA physical status III–IV (24% vs. 11%; *p* = 0.003). At baseline (T_0_, after patient conditioning), the PIH group exhibited a lower MAP (102 vs. 106 mmHg; *p* = 0.003) and heart rate (72 vs. 77 bpm; *p* = 0.008). The cumulative propofol induction dose (mg/kg actual body weight) before the first MAP < 65 mmHg was lower in the PIH group (2.0 vs. 2.1 mg/kg; *p* = 0.014). There were no statistically significant differences between groups in sex, body mass index, comorbidities, chronic medications, haemoglobin, creatinine, glucose, electrolytes, fasting duration, pre-induction fluid administration, induction doses of fentanyl and rocuronium, or surgical procedure (all *p* > 0.05; Table 1 and Supplementary Tables S1–S2). Thoracic surgery was numerically more frequent in the PIH group, although the difference was not statistically significant.

**Table 1:** Patient baseline clinical and intra-operative characteristics

Characteristics	All (*n* = 405)	No PIH (*n* = 244)	PIH (*n* = 161)	*p*
Female	233 (58)	137 (56)	96 (60)	0.488
Age (years)	57 [47–63]	55 [45–62]	59 [51–63]	**0.003**
BMI (kg/m^2^)	23 [21–25]	23 [21–25]	23 [21–25]	0.763
ASA physical status				
I	90 (22)	62 (25)	28 (17)	**0.003**
II	249 (61)	154 (63)	95 (59)	
III–IV	66 (16)	28 (11)	38 (24)	
Current smoking	28 (6.9)	15 (6.1)	13 (8.1)	0.454
Chemotherapy	3 (0.7)	1 (0.4)	2 (1.2)	0.566
Hypertension	183 (45)	113 (46)	70 (43)	0.575
ACEIs or ARBs	69 (17)	36 (15)	33 (20)	0.132
Beta blocker	43 (11)	22 (9)	21 (13)	0.198
Calcium channel blockers	117 (29)	75 (31)	42 (26)	0.312
Diuretics	12 (3.0)	7 (2.9)	5 (3.1)	> 0.999
HMSD	52 (13)	33 (14)	19 (12)	0.612
Chronic coronary syndrome	16 (4.0)	8 (3.3)	8 (5.0)	0.393
Diabetes mellitus	62 (15)	34 (14)	28 (17)	0.344
Chronic obstructive pulmonary disease	1 (0.2)	1 (0.4)	0 (0)	> 0.999
Peripheral arterial disease	2 (0.5)	1 (0.4)	1 (0.6)	> 0.999
Stroke	6 (1.5)	3 (1.2)	3 (1.9)	0.686
Chronic kidney disease	11 (2.7)	4 (1.6)	7 (4.3)	0.123
Cirrhosis	4 (1.0)	3 (1.2)	1 (0.6)	> 0.999
Cushing	8 (2.0)	4 (1.6)	4 (2.5)	0.718
Hyperthyroidism	5 (1.2)	3 (1.2)	2 (1.2)	> 0.999
Hypothyroidism	5 (1.2)	4 (1.6)	1 (0.6)	0.652
MAP_T0_ (mmHg)	105 ± 14	106 ± 13	102 ± 14	**0.003**
HR_T0_ (bpm)	75 [67–84]	77 [68–85]	72 [65–82]	**0.008**
Liquid fasting (h)	10 [6–12]	10 [6–12]	10 [6–12]	0.368
Solid fasting (h)	16 [13–20]	16 [13–19]	16 [14–20]	0.711
Pre-induction fluid administration (ml/kg/h)	0.94 [0.71–1.47]	0.93 [0.70–1.51]	0.94 [0.72–1.37]	0.809
Propofol (mg/kg)	2.0 [1.8–2.3]	2.1 [1.8–2.3]	2.0 [1.7–2.2]	**0.014**
Fentanyl (µg/kg)	2.1 [1.9–2.5]	2.1 [1.9–2.5]	2.2 [1.9–2.5]	0.947
Rocuronium (mg/kg)	0.7 [0.6–0.8]	0.7 [0.6–0.8]	0.7 [0.6–0.8]	0.294

Values are *n* (%), median [IQR] or mean ± SD, significant *p*-values in bold

*ASA* American Society of Anesthesiologists, *BMI* body mass index, *HR* heart rate, *MAP* mean arterial pressure, *PIH* post-induction hypotension, *T*_*0*_ post-patient conditioning in the operating threate, *ACEIs* or *ARBs* angiotensin-converting enzyme inhibitors or angiotensin II receptor blockers, HMSD, pre-operative antihypertensive medication (morning dose)

As shown in Table 2, the PIH group had a lower left ventricular mass index (LVMI) (79 vs. 83 g/m^2^; *p* = 0.022) and a higher prevalence of an *E*_a_/*E*_es_ ratio > 1 (14% vs. 7.8%; *p* = 0.036). Echocardiographic estimates of aortic mechanical properties did not differ significantly between groups (all *p* > 0.05).

**Table 2:** Pre-operative echocardiographic and ventriculo-arterial coupling variables

Echocardiographic variables	All (*n* = 405)	No PIH (*n* = 244)	PIH (*n* = 161)	*p*
IVSD (mm)	9.2 [8.0–10.4]	9.3 [8.0–10.6]	9.0 [8.0–10.3]	0.292
LVIDd (mm)	46.0 [42.9–49.0]	46.3 [43.0–49.0]	45.6 [42.5–49.1]	0.397
Grade 1 diastolic dysfunction	55 (14)	35 (14)	20 (12)	0.581
LV hypertrophy	43 (11)	26 (11)	17 (11)	0.975
*E/A* ratio	0.8 [0.7–1.1]	0.8 [0.7–1.1]	0.8 [0.7–1.1]	0.522
e′ (cm/s)	8.6 [7.0–10.4]	8.6 [6.9–10.5]	8.6 [7.1–10.1]	0.258
*E*/e′ ratio	7.6 [6.5–9.1]	7.6 [6.5–9.0]	7.6 [6.3–9.2]	0.821
S′ (cm/s)	12.2 [10.8–13.4]	12.2 [10.9–13.5]	12.1 [10.7–13.2]	0.738
TAPSE (mm)	22.6 [20.2–25.2]	22.6 [20.4–25.3]	22.5 [20.0–25.0]	0.576
LA volume index (ml/m)	30 [26–35]	30 [26–35]	31 [26–36]	0.804
LV mass index (g/m^2^)	81 [70–96]	83 [71–98]	79 [66–94]	**0.022**
LV EF Simpson (%)	61.1 [59.0–63.1]	61.1 [59.0–63.3]	61.1 [59.0–63.1]	0.744
LV GLS (%)	−17.9 [−19.3 to −16.4]	−18.0 [−19.3 to −16.5]	−17.8 [−19.4 to −16.4]	0.464
LV ESV index (ml/m^2^)	20.8 [18.7–24.0]	20.9 [18.6–24.2]	20.7 [18.7–23.9]	0.549
LV EDV index (ml/m^2^)	55 [48–62]	55 [48–62]	55 [49–61]	0.896
*CI* (l/min/m^2^)	2.7 [2.3–3.2]	2.7 [2.4–3.2]	2.6 [2.3–3.1]	0.053
*E*_es_ (mmHg/ml)	2.4 [2.1–3.0]	2.5 [2.0–3.0]	2.4 [2.1–2.9]	0.717
*E*_a_ (mmHg/ml)	1.9 [1.6–2.2]	1.9 [1.6–2.2]	1.9 [1.7–2.2]	0.994
*E*_a_/*E*_es_ ratio > 1	42 (10)	19 (7.8)	23 (14)	**0.036**
Aortic stiffness index	7 [4–12]	6 [4–12]	7 [4–12]	0.552
Aortic strain (%)	7.1 [3.8–11.1]	7.5 [3.6–11.3]	6.8 [3.9–10.8]	0.714
Aortic distensibility (cm^2^ /dyn × 10^–6^)	2.2 [1.2–3.8]	2.3 [1.2–3.8]	2.2 [1.2–3.8]	0.865

Values are *n* (%) or median [IQR]; significant *p*-values in bold

*CI* cardiac index, *E/A* ratio of early to late diastolic mitral inflow velocities, e′ early diastolic mitral annular velocity, *E*_a_ effective arterial elastance, *EDV* end-diastolic volume, *E*_es_ left ventricular end-systolic elastance, *EF* ejection fraction, *ESV* end-systolic volume, *GLS* global longitudinal strain, *IVSd* interventricular septal thickness in diastole, *LA* left atrium, *LV* left ventricle, *LVIDd* left ventricular internal diameter in diastole, *PIH* post-induction hypotension, *S*′ systolic tricuspid annular velocity, *TAPSE* tricuspid annular plane systolic excursion

### Predictors associated with PIH

**Fig. 2:**
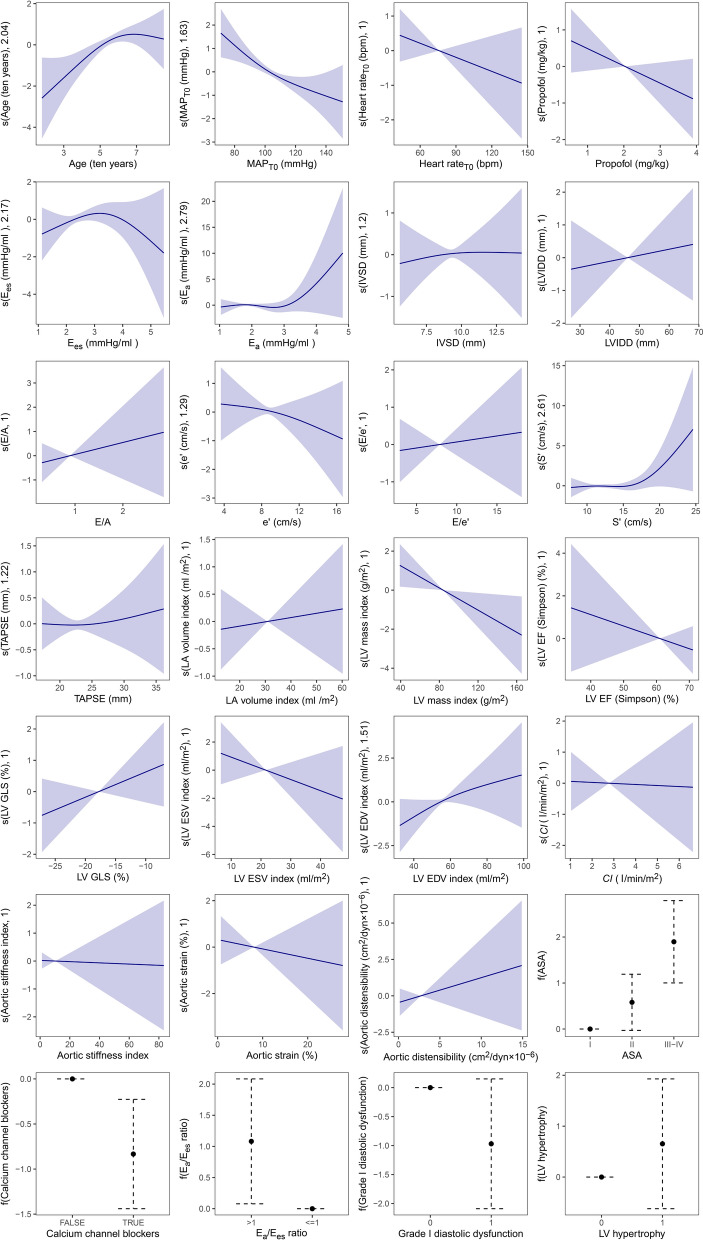
Association of risk factors with log odds ratio of PIH in the M_full_ model. The association between these risk factors (shown on the *x*-axis) and the log odds ratio of post-induction hypotension (PIH) (shown on the *y*-axis) was analysed using a GAM (generalised additive model). M_full_ model: six clinical variables (via LASSO), 19 echocardiographic measurements, and three VAC-related variables (continuous *E*_a_ and *E*_es_, and *E*_a_/*E*_es_ ratio > 1). *ASA* American Society of Anesthesiologists, *CCBs* calcium channel blockers, *CI* cardiac index, *E/A* ratio of early to late diastolic mitral inflow velocities, e′ early diastolic mitral annular velocity, *E*_a_ effective arterial elastance, *EDV* end-diastolic volume, *E*_es_ left ventricular end-systolic elastance, *EF* ejection fraction, *ESV* end-systolic volume, *GLS* global longitudinal strain, *IVSd* interventricular septal thickness in diastole, *LA* left atrium, *LV* left ventricle, *LVIDd* left ventricular internal diameter in diastole, *MAP* mean arterial pressure, *PEP* pre-ejection period, *S*′ systolic tricuspid annular velocity, *T*_*0*_ post-patient conditioning in the operating theatre, *TAPSE* tricuspid annular plane systolic excursion, *TSP* total systolic period

In the M_full_ model, GAM identified several predictors of PIH (Fig. 2 and Supplementary Table S3). Higher ASA physical status (III–IV) was strongly associated with increased PIH risk (OR 6.66; 95% CI 2.72–16.3; *p* < 0.001). An *E*_a_/*E*_es_ ratio > 1 was also significantly associated with increased PIH risk (OR 2.95; 95% CI 1.08–8.03; *p* = 0.034), as was advancing age (per decade; *p* = 0.010).

Among echocardiographic variables, a greater LVMI was associated with a lower risk of PIH (*p* = 0.023). In addition, use of calcium channel blockers (CCBs) (OR 0.43; 95% CI 0.24–0.80; *p* = 0.007) and higher baseline MAP (*p* = 0.002) were both associated with reduced PIH risk.

*E*_es_ and *E*_a_ demonstrated non-linear relationships with PIH risk; however, these associations were not statistically significant (*p* > 0.05). No other variables in the M_full_ model were significantly associated with PIH.

### Added predictive value of VAC and echocardiography for PIH

Added value was reflected by AUC and FNI. Compared with M_0_ (AUC, 0.720; 95% CI 0.669–0.771), M_echo_ increased the AUC to 0.768 (95% CI 0.720–0.816; Holm-adjusted* p* = 0.018) and yielded a 37% FNI (Holm-adjusted *p* = 0.199). M_full_ increased the AUC to 0.785 (95% CI 0.739–0.831; Holm-adjusted* p* = 0.002) and yielded a 46% FNI compared with M_0_ (Holm-adjusted* p* = 0.030) and a 14% FNI compared with M_echo_ (Holm-adjusted* p* = 0.018). However, the ΔAUC compared with M_echo_ was not statistically significant (0.017; 95% CI −0.004–0.039; Holm-adjusted* p* = 0.199) (Tables 3–4).

**Table 3:** Comparison of area under the curve (AUC) and incremental gains between models

Model	AUC (95% CI)	ΔAUC (95% CI)	Unadjusted* p*	Holm-adjusted* p*
M_0_	0.720 (0.669–0.771)	–	−	−
M_echo_	0.768 (0.720–0.816)	0.048 (0.016–0.081)	**0.0036**	**0.018**
M_full_	0.785 (0.739–0.831)	0.065 (0.030–0.101)	**0.0003**	**0.002**
M_full_ vs M_echo_	–	0.017 (−0.004–0.039)	0.1166	0.199

Adjusted *p*-values were calculated for multiple comparisons using Holm’s method; statistically significant *p*-values are shown in bold

Pairwise comparisons of the area under the curve between models were performed using DeLong’s test

M_0_ includes six clinical variables selected via LASSO regression from a broad set of clinical and laboratory variables; M_echo_ adds 19 echocardiographic measurements; M_full_ builds on M_echo_ by adding *E*_a_ and *E*_es_ (continuous) and an *E*_a_/*E*_es_ ratio > 1

**Table 4:** Added predictive value of VAC and echocardiography in predicting PIH

Model comparison	M_echo_ vs M_0_	M_full_ vs M_0_	M_full_ vs M_echo_
Fraction of new information	0.37	0.46	0.14
Degrees of freedom	24.97	29.05	4.09
Deviance	34.36	49.63	15.27
Unadjusted *p*	0.1	**0.01**	** < 0.001**
Holm-adjusted *p*	0.199	**0.030**	**0.018**

Differences in deviance were assessed with likelihood-ratio tests

Adjusted *p* were calculated with the Holm correction; significant *p*-values in bold

M_0_ includes six clinical variables selected via LASSO regression from a broad set of clinical and laboratory variables; M_echo_ adds 19 echocardiographic measurements; M_full_ builds on M_echo_ by adding *E*_a_ and *E*_es_ (continuous) and an *E*_a_/*E*_es_ ratio > 1

*PIH* post-induction hypotension, *VAC* ventriculo-arterial coupling

### Contribution of variables in the full model

In the M_full_ model, the largest contributors to the relative explained variance in PIH risk were ASA class III–IV (15.27%; 95% CI 1.98–15.58), baseline MAP (8.75%; 95% CI 1.08–12.85), age per decade (7.35%; 95% CI 0.29–11.69), and CCB use (6.82%; 95% CI 0.07–9.84). Echocardiographic measurements and VAC-related variables showed smaller contributions, including LVMI (4.61%; 95% CI 0.36–10.08), left ventricular end-diastolic volume index (3.37%; 95% CI 0.21–13.08), E_a_ (3.80%; 95% CI 0.07–7.20), *E*_es_ (2.69%; 95% CI 0.08–8.63) and an *E*_a_/*E*_es_ ratio > 1 (3.86%; 95% CI 0.01–6.84). All other variables accounted for smaller proportions of the relative explained variance (Supplementary Fig. S1).

## Discussion

This study found that older age, ASA III–IV status, lower baseline MAP, higher LV mass index, and an *E*_a_/*E*_es_ ratio > 1 were independently associated with increased PIH risk, whereas calcium channel blocker use was associated with lower odds of PIH. Adding echocardiographic variables (M_echo_) significantly improved discrimination, as reflected by an increase in AUC compared with the clinical model (M_0_) but did not yield a statistically significant gain in FNI. The further addition of VAC-related variables (M_full_) resulted in significant increases in both AUC and FNI compared with M0, and provided a statistically significant incremental gain in FNI, but not ΔAUC, when compared with M_echo_.

### The role of ventriculo-arterial uncoupling in PIH

This study found that impaired ventriculo-arterial coupling, defined as an *E*_a_/*E*_es_ ratio > 1, was independently associated with an increased risk of PIH in the GAM analysis. VAC reflects the interaction between left ventricular contractility and arterial load; when coupling is impaired, the cardiovascular system operates closer to its haemodynamic limits, reducing the heart’s capacity to accommodate the haemodynamic effects of anaesthetic agents, increasing susceptibility to hypotension [12]. Although the present study did not directly assess the dynamic effects of anaesthetic agents on VAC, prior investigation indicates that propofol exerts little direct effect on VAC, whereas sevoflurane may increase *E*_a_ and decrease *E*_es_, promoting ventriculo-arterial uncoupling [16]. Accordingly, hypotension during propofol induction is generally attributed to predominant vasodilation rather than primary depression of myocardial contractility [26].

The threshold *E*_a_/*E*_es_ ratio of 1 reflects the physiological balance between *E*_a_ and *E*_es_: when *E*_a_ increases, *E*_es_ must increase proportionally to preserve VAC. Failure of this compensatory mechanism results in uncoupling with consequent reduction in mechanical efficiency [9, 27]. An *E*_a_/*E*_es_ ratio > 1 has been proposed as a clinically meaningful marker of impaired coupling, particularly in critical care populations [28]. A physiological range of 1 ± 0.36 is frequently cited [12]; however, this reference range originates from an invasive haemodynamic study conducted in a relatively small cohort [29].

GAM demonstrated a non-linear association between both components of VAC (*E*_a_ and *E*_es_) and PIH risk. Although an increase in *E*_a_ was associated with a higher likelihood of PIH, this association did not reach statistical significance and may reflect chronic elevation of arterial afterload, which is commonly seen in older patients and those with hypertension or diabetes. In these populations, reduced arterial compliance—reflecting increased arterial stiffness—leads to higher *E*_a_ values. Effective arterial elastance is a composite index of arterial load; *E*_a_ integrates the effects of peripheral resistance, pulsatile arterial elastance, ejection time, and the diastolic pressure decay constant [12].

A previous study has established an independent link between increased arterial stiffness and PIH [30]. To preserve VAC under such conditions, the left ventricle progressively increases contractility. Sustained compensatory demand may deplete cardiac reserve and predispose to heart failure with preserved ejection fraction (HFpEF). In HFpEF, parallel increases in *E*_a_ and *E*_es_ produce a nominally normal *E*_a_/*E*_es_ ratio, potentially masking functional uncoupling [31] and thereby increasing vulnerability to haemodynamic instability during anaesthesia.

Yildirim et al*.* reported a three-fold increase in the risk of PIH among patients with a pre-induction *E*_a_ exceeding 1.08 mmHg/ml/m^2^. However, this threshold is challenging to apply in individual clinical practice, as it is indexed to body surface area. These authors also observed similar post-induction *E*_a_ values between PIH and non-PIH groups, despite higher pre-induction *E*_a_ values in the PIH cohort [18]. This pattern suggests a greater relative reduction in *E*_a_ following induction and supports increased sensitivity to the vasodilatory effects of anaesthetic agents.

In the present study, pre-operative measures of systolic function and cardiac performance, including EF, GLS, end-systolic volume (ESV), end-diastolic volume (EDV), and cardiac index (*CI*) did not differ between groups, despite the theoretical expectation that elevated *E*_a_ would reduce ESV and increase EDV in patients with PIH. GAM analysis demonstrated a linear association between higher EF, greater absolute GLS, higher ESV, and higher *CI* and lower PIH risk, whereas increased EDV showed a non-linear trend towards higher risk; however, none of these associations achieved statistical significance.

*E*_es_ showed an inverted U-shaped association with PIH risk, with lower risk observed at both lower and higher values. One hypothesis is that higher *E*_es_ reflects preserved ventricular contractile reserve, conferring greater tolerance to anaesthesia induction. At lower *E*_es_ values, risk of PIH may depend on the prevailing arterial load: lower *E*_a_ may preserve coupling, whereas normal or higher *E*_a_ may reflect relative uncoupling and greater susceptibility to PIH. Because *E*_a_ and *E*_es_ represent distinct components of cardiovascular function, interpreting either alone may fail to capture the haemodynamic context. In contrast, the *E*_a_/*E*_es_ ratio integrates both elements, providing a physiologically meaningful assessment of cardiovascular reserve [12, 27].

### Clinical, echocardiographic, and pharmacological predictors of PIH

The present findings identified advanced age and ASA III–IV status as significant predictors of PIH, consistent with prior studies [32, 33]. Chronic CCB use was associated with a lower risk of PIH, corroborating previous findings [8, 34], although the underlying mechanism remains uncertain. While evidence regarding beta-blocker use remains inconclusive, ACEIs/ARBs are well-recognised risk factors for PIH, particularly long-acting agents [8, 34].

Echocardiographic examinations showed that LVMI was significantly lower in the PIH group, and PIH risk increased linearly with decreasing LVMI, despite all values being within the normal range. This finding may reflect reduced ventricular reserve. Hypertension, a major cause of left ventricular hypertrophy and elevated LVMI [35, 36], was equally prevalent between groups, and baseline MAP was higher in the non-PIH group. Higher baseline MAP appeared protective, likely reflecting a threshold effect related to defining PIH as MAP < 65 mmHg, whereby a higher starting MAP reduces the likelihood of crossing the absolute cut-off rather than indicating causality. In contrast, studies defining hypotension by relative reductions in MAP have reported higher baseline MAP as a risk factor [37]. Baseline MAP also correlated with LVMI, suggesting potential collinearity and that LVMI may not be independently protective. All diastolic dysfunction was grade 1, which may partly explain the lack of association between diastolic indices and PIH despite prior reports [8]. Regional wall motion abnormalities, previously identified as a potential risk factor [8], were rare in this cohort, thereby precluding meaningful analysis.

In the present study, the cumulative propofol induction dose indexed to actual body weight before the first recorded PIH event was lower in the PIH group, whereas fentanyl and rocuronium doses did not differ between groups. Propofol may precipitate hypotension via sympathetic and baroreflex inhibition with reduced systemic vascular resistance [38], and higher induction doses have been linked to PIH [32]. In this study, propofol was titrated to a BIS target using an institutional protocol with supplemental boluses as needed, allowing individualised dosing. The lower propofol dose in the PIH group should therefore be interpreted cautiously and may reflect individualised titration, whereby older or frailer patients may reach the BIS target with less propofol yet remain more haemodynamically vulnerable, whereas younger or fitter patients require more propofol but are less prone to PIH [39]. Because no syringe pump was used, the exact drug administration rate and the time required to administer induction agents could not be reliably quantified, thereby limiting between-group comparisons.

### The added value of VAC in PIH risk stratification

Recent PIH prediction models developed using conventional regression or machine-learning approaches with diverse pre-operative data (clinical factors, medications, and echocardiographic or haemodynamic measures) have shown only modest performance (AUC: 0.72–0.84), highlighting PIH’s multifactorial pathophysiology and the challenge of capturing key haemodynamic mechanisms [3, 4, 40].

In the present study, adding echocardiographic measurements to the baseline clinical model (M_0_) significantly increased AUC but yielded a non-significant FNI of 37%. Adding VAC-related variables significantly increased AUC and contributed significant gains in FNI, yielding 46% compared with M_0_ and 14% compared with M_echo_, despite a non-significant increase in AUC compared with M_echo_. An additional 14% FNI means that, among the information used to predict PIH, one-seventh was uniquely contributed by VAC-related variables. These findings suggest that combining VAC-related variables with a clinical–echocardiographic model may improve PIH prediction, an effect captured more clearly by FNI than by pure increase in AUC. This aligns with Harrell’s recommendation that likelihood-ratio testing is statistically more efficient than AUC for quantifying the added value of predictors [41].

### Relative explained variance in M_full_

Relative explained variance helped to place these findings in context. Model performance was driven primarily by clinical factors, particularly ASA class III–IV, baseline MAP, age, and CCB use. Notably, beyond these dominant clinical contributors, only a small number of echocardiographic variables showed meaningful contributions, with LVMI and VAC-related variables standing out. Among the many echocardiographic variables evaluated, VAC-related variables were more influential than most others. Notably, an *E*_a_/*E*_es_ ratio > 1 contributed slightly more than either *E*_a_ or *E*_es_ alone, suggesting that coupling captures predictive information not fully reflected by its individual components. This pattern aligns with the main results, in which an *E*_a_/*E*_es_ ratio > 1 was independently associated with PIH, and the incorporation of VAC-related variables improved prediction beyond the clinical model, with statistically significant gains in both AUC and FNI.

### Limitations and future directions

This study has several important limitations that merit consideration. Pre-operative echocardiography performed within 48 h may not fully reflect haemodynamics at induction. The number of candidate predictors was large relative to the sample size; although LASSO reduced model complexity, variable selection may have introduced bias in performance estimates and the apparent added value of echocardiographic and VAC variables. This single-centre cohort had largely normal echocardiographic findings, which may limit generalisability. PIH was defined by an absolute MAP threshold, potentially making higher baseline MAP appear protective due to a threshold effect. Finally, non-invasive blood pressure measured every 2 min may miss brief hypotension and limit the assessment of hypotension duration or burden.

### Clinical implications

From a translational perspective, VAC assessment may provide incremental benefit but must be weighed against training requirements and cost. The added complexity and operator dependence associated with VAC measurement may, however, be mitigated by the integration of automated quantification through AI-driven software. A pragmatic approach is therefore to embed VAC assessment within clinically indicated pre-operative echocardiography, requiring only minimal additional measurements that are routinely obtainable, including SV from VTI and LVOT diameter, PEP, TSP, and NIBP. For experienced echocardiographers, this approach is likely to be feasible with limited additional training. In this study, we included a heterogeneous population, including asymptomatic and clinically stable individuals. According to current guidelines, pre-operative echocardiography should be reserved for patients with new dyspnoea or suspected ventricular dysfunction (Class 1), or for those with known heart failure and a change in clinical status (Class 2a) [42]. Therefore, our findings should be interpreted in the context of contemporary guidelines on perioperative cardiovascular management for non-cardiac surgery. In patients without a history of cardiac disease or cardiac-related symptoms, routine pre-operative echocardiography is not recommended and should be considered exploratory pending further supporting evidence.

In patients considered at high risk, VAC can inform anticipatory management before induction, supporting tailored agent selection and electroencephalography-guided titration, more frequent NIBP cycling (every 1–2 min), consideration of pre-induction invasive blood pressure monitoring and, in selected cases, advanced haemodynamic monitoring, with vasoactive drugs readily available and used proactively when clinically appropriate. These suggestions are hypothesis-generating and should be evaluated prospectively. Accordingly, future multicentre studies in more heterogeneous populations are needed to validate these findings and to determine whether VAC-informed perioperative strategies can reduce PIH and improve clinical outcomes.

## Conclusions

An *E*_a_/*E*_es_ ratio > 1 was independently associated with post-induction hypotension, highlighting impaired ventriculo-arterial coupling as a marker of haemodynamic vulnerability during anaesthesia induction. Although adding VAC-related variables did not significantly increase the AUC, it provided meaningful new information beyond a clinical–echocardiographic model, suggesting improved risk stratification through complementary physiological assessment. Integration of non-invasive VAC assessment into clinically indicated echocardiography may improve pre-operative risk stratification for PIH in non-cardiac surgery. Further multicentre studies are needed to validate these findings and to explore whether VAC-guided perioperative management can improve haemodynamic stability and patient outcomes.

## Supplementary Information

Supplementary Material 1.

Supplementary Material 2.

## Data Availability

The datasets supporting this study are included in the published article. Additional information is available from the corresponding author upon reasonable request. The statistical analysis code is openly accessible at https://github.com/trinhdhk/VAC-PIH.

## Abbreviations

ASA: American Society of Anesthesiologists
ACEIs: Angiotensin-converting enzyme inhibitors
ARBs: Angiotensin II receptor blockers
AUC: Area under the receiver operating characteristic curve
BMI: Body mass index
CCB: Calcium channel blockers
*CI*: Cardiac index
CI: Confidence interval
E/A: Ratio of early to late diastolic mitral inflow velocities
e′: Early diastolic mitral annular velocity
*E*_a_: Effective arterial elastance
EDV: End-diastolic volume
*E*_es_: Left ventricular end-systolic elastance
EF: Ejection fraction
ESV: End-systolic volume
FNI: Fraction of new information
GAM: Generalised additive model
GLS: Global longitudinal strain
HR: Heart rate
IQR: Interquartile range
IVSd: Interventricular septal thickness in diastole
LA: Left atrium
LASSO: Least absolute shrinkage and selection operator
LV: Left ventricle
LVIDd: Left ventricular internal diameter in diastole
LVOT: Left ventricular outflow tract diameter
MAP: Mean arterial pressure
NIBP: Non-invasive blood pressure
OR: Odds ratio
PIH: Post-induction hypotension
S′: Systolic tricuspid annular velocity
SD: Standard deviation
T_0_: Post-patient conditioning in the operating theatre
TAPSE: Tricuspid annular plane systolic excursion
TTE: Transthoracic echocardiography
VAC: Ventriculo-arterial coupling
VTI: Velocity–time integral
