# Supplementary material for: Predictive value of ventriculo-arterial coupling for hypotension after induction of anaesthesia: a prospective observational cohort study
Source: Intensive Care Med Exp. 2026 Apr 21;14:50. doi: 10.1186/s40635-026-00899-0 (PMC13100084; doi:10.1186/s40635-026-00899-0)
Supplement: Supplementary file 1 — Supplementary Material 1. [file 40635_2026_899_MOESM1_ESM.docx]

**Supplementary Data**

**Predictive value of ventriculo-arterial coupling for hypotension after induction of anesthesia: A prospective observational cohort study**

**Tables**

**1. Supplementary Table S1** Pre-operative laboratory parameters

**2. Supplementary Table S2** Types of surgery

**3. Supplementary Table S3** Multivariable GAM analysis of risk factors for PIH in M_full_ model

**Figures**

**1. Supplementary Fig. S1** Relative explained variance of each variable in PIH prediction in M_full_ model.

**Supplementary Methods**

**1.** **Supplementary Method S1** Calculation of fraction of new information (FNI)

**Supplementary Table S1** Pre-operative laboratory parameters

| Laboratory parameters | All (*n*=405) | No PIH (*n*=244) | PIH (*n*=161) | *p* |
| --- | --- | --- | --- | --- |
| Haemoglobin (g/dl) | 13.4 ± 1.5 | 13.5 ± 1.5 | 13.3 ± 1.6 | 0.110 |
| Creatinine (µmol/l) | 68 [58–81] | 68 [58–80] | 69 [58–82] | 0.487 |
| Glucose (mmol/l) | 5.0 [4.5–5.6] | 5.0 [4.5–5.5] | 5.0 [4.5–5.7] | 0.418 |
| Sodium (mmol/l) | 140 [139–142] | 140 [139–142] | 140 [139–141] | 0.558 |
| Potassium (mmol/l) | 3.8 [3.6–4.0] | 3.8 [3.6–4.0] | 3.8 [3.6–4.0] | 0.512 |
| Chloride (mmol/l) | 104 [102–106] | 104 [102–106] | 104 [103–106] | 0.266 |
| Calcium (mmol/l) | 2.2 [2.2–2.3] | 2.2 [2.2–2.3] | 2.2 [2.2–2.3] | 0.911 |

Values are median [IQR] or mean ± SD. PIH, post-induction hypotension.

**Supplementary Table S2** Types of surgery

| Types of surgery | All (*n*=405) | No PIH (*n*=244) | PIH (*n*=161) | *p* |
| --- | --- | --- | --- | --- |
| Breast surgery | 14 (3.5) | 10 (4.1) | 4 (2.5) | 0.315 |
| Gastrointestinal surgery | 28 (6.9) | 16 (6.6) | 12 (7.5) |  |
| Hepatobiliary surgery | 71 (18) | 44 (18) | 27 (17) |  |
| Neurosurgery | 12 (3.0) | 5 (2.0) | 7 (4.3) |  |
| Orthopedic surgery | 72 (18) | 46 (19) | 26 (16) |  |
| ENT surgery | 51 (13) | 27 (11) | 24 (15) |  |
| Spine surgery | 37 (9.1) | 22 (9.0) | 15 (9.3) |  |
| Thoracic surgery | 11 (2.7) | 3 (1.2) | 8 (5.0) |  |
| Thyroid surgery | 51 (13) | 34 (14) | 17 (11) |  |
| General surgery | 5 (1.2) | 2 (0.8) | 3 (1.9) |  |
| Urology surgery | 51 (13) | 33 (14) | 18 (11) |  |
| Vascular surgery | 2 (0.5) | 2 (0.8) | 0 (0) |  |

Values are *n* (%). PIH, post-induction hypotension; ENT, ear, nose, and throat.

**Supplementary Table S3** Multivariable GAM analysis of risk factors for PIH in M_full_ model

| Variables | OR | 95% CI | *p* |
| --- | --- | --- | --- |
| ASA II | 1.79 | 0.97–3.29 | 0.062 |
| ASA III–IV | 6.66 | 2.72–16.3 | **<0.001** |
| E_a_/E_es_ ratio >1 | 2.95 | 1.08–8.03 | **0.034** |
| LV hypertrophy | 1.92 | 0.54–6.88 | 0.315 |
| Calcium channel blockers | 0.43 | 0.24–0.80 | **0.007** |
| Grade 1 diastolic dysfunction | 0.38 | 0.12–1.16 | 0.090 |
| Age (ten years) |  |  | **0.010** |
| MAP_T0_ (mmHg) |  |  | **0.002** |
| Heart rate at T_0_ (bpm) |  |  | 0.253 |
| Propofol (mg/kg) |  |  | 0.113 |
| E_es_ (mmHg/ml) |  |  | 0.341 |
| E_a_ (mmHg/ml) |  |  | 0.416 |
| IVSD (mm) |  |  | 0.862 |
| LVIDD (mm) |  |  | 0.643 |
| E/A ratio |  |  | 0.479 |
| e’ (cm/s) |  |  | 0.602 |
| S’ (cm/s) |  |  | 0.323 |
| TAPSE (mm) |  |  | 0.866 |
| LA volume index (ml/m^2^) |  |  | 0.704 |
| LV mass index (g/m^2^) |  |  | **0.023** |
| LV EF Simpson (%) |  |  | 0.346 |
| LV GLS (%) |  |  | 0.206 |
| LV ESV index (ml/m^2^) |  |  | 0.286 |
| LV EDV index (ml/m^2^) |  |  | 0.193 |
| *CI* (l/min/m^2^) |  |  | 0.904 |
| Aortic stiffness index |  |  | 0.893 |
| Aortic strain (%) |  |  | 0.578 |
| Aortic distensibility (cm^2^/dyn x 10^-6^) |  |  | 0.360 |

M_full_ model: six clinical variables (via LASSO), 19 echocardiographic measurements, and three VAC-related variables (Continuous E_a_ and E_es_, and E_a_/E_es_ ratio >1). Categorical variables are shown as OR (95% CI). Continuous variables were modeled non-linearly and shown as spline plots; thus, a single OR is not reported because effects vary across the predictor range (see Fig. 2). Significant *p*-values in bold.

ASA, American Society of Anesthesiologists; *CI*, cardiac index; E/A, ratio of early to late diastolic mitral inflow velocities; e’, early diastolic mitral annular velocity; E_a_, effective arterial elastance; EDV, end-diastolic volume; E_es_, left ventricular end-systolic elastance; EF, ejection fraction; ESV, end-systolic volume; GAM, generalised additive model; GLS, global longitudinal strain; IVSd, interventricular septal thickness in diastole; LA, left atrium; LV, left ventricle; LVIDd, left ventricular internal diameter in diastole; MAP, mean arterial pressure; PEP, pre-ejection period; PIH, post-induction hypotension; S’, systolic tricuspid annular velocity; T_0_, post-patient conditioning in the operating theatre; TAPSE, tricuspid annular plane systolic excursion; TSP, total systolic period.

**Insert Supplementary Fig. S1 here**

**Supplementary Fig. S1** Relative explained variance of each variable in PIH prediction in M_full_ model

M_full_ model: six clinical variables (via LASSO), 19 echocardiographic measurements, and three VAC-related variables (Continuous E_a_ and E_es_, and E_a_/E_es_ ratio >1).

ASA, American Society of Anesthesiologists; CCBs, calcium channel blockers; *CI*, cardiac index; E/A, ratio of early to late diastolic mitral inflow velocities; e’, early diastolic mitral annular velocity; E_a_, effective arterial elastance; EDV, end-diastolic volume; E_es_, left ventricular end-systolic elastance; EF, ejection fraction; ESV, end-systolic volume; GLS, global longitudinal strain; IVSd, interventricular septal thickness in diastole; LA, left atrium; LV, left ventricle; LVIDd, left ventricular internal diameter in diastole; MAP, mean arterial pressure; PEP, pre-ejection period; S’, systolic tricuspid annular velocity; T_0_, post-patient conditioning in the operating theatre; TAPSE, tricuspid annular plane systolic excursion; TSP, total systolic period.

**Supplementary Methods S1: Calculation of fraction of new information (FNI)**

**Models**
Added predictive value was assessed across three nested models: (1) M_0_, including clinical and laboratory variables selected by LASSO; (2) M_echo_, adding echocardiographic measurements; and (3) M_full_, further incorporating E_a_ and E_es_ (continuous) and an E_a_/E_es_ ratio >1.

**Fraction of new information (FNI)**

Added value was summarised using the FNI, defined via likelihood-ratio (LR) statistics. Let Mnull denote the intercept-only model and let ℓ(M) be the maximised log-likelihood of model M. Define LR(M) = 2 × [ℓ(M) − ℓ(Mnull)]. For a nested comparison Mpre ⊂ Mpost, the adequacy index (AI) is AI = LR(Mpre)/LR(Mpost), representing the proportion of total model information explained by the pre-model. The fraction of new information is FNI = 1 − AI = 1 − LR(Mpre)/LR(Mpost). We report FNI for M_0_ → M_echo_ and M_echo_ → M_full_ (and, where relevant, M_0_ → M_full_).

**Statistical testing and multiplicity**

Added value was tested using likelihood-ratio tests for nested models (M_0_ vs M_echo_; M_echo_ vs M_full_; and M_0_ vs M_full_). Prespecified p-values were Holm-adjusted for multiple comparisons, and both unadjusted and adjusted p-values were reported.
